# The past and future of RRI

**DOI:** 10.1186/s40504-014-0017-4

**Published:** 2014-11-06

**Authors:** Arie Rip

**Affiliations:** University of Twente, P.O. Box 217, Enschede, 7500AE Netherlands

**Keywords:** Responsibility, Division of moral labour, Social innovation, Path dependency, Reflection

## Abstract

Within the space of a few years, the idea of Responsible Research and Innovation, and its acronym RRI, catapulted from an obscure phrase to the topic of conferences and attempts to specify and realize it. How did this come about, and against which backdrop? What are the dynamics at present, and what do these imply for the future of RRI as a discourse, and as a patchwork of practices? It is a social innovation which creates opening in existing (and evolving) divisions of moral labour, a notion that is explained with the help of the history of responsibility language. It is filled in for the present situation and ongoing developments. Some elements may stabilize and this creates a path into the future. There will be reductions of the originally open-ended innovation, some productive, others less so. This is a reason to regularly inquire into the value of the reductions and the directions the path is taking.

## Background

Within the space of a few years, the idea of Responsible Research and Innovation, and its acronym RRI, catapulted from an obscure phrase to an issue in the European Commission’s Horizon 2020 Program, to a topic of conferences (Danish EU Presidency 2012) and edited volumes (Owen et al. 2013a). Without using the exact term, a discourse on responsible development of nanotechnology was visible already in the mid 2000s, and nanotechnology can be seen as the lead domain for discourse and activities on RRI^a^.

It is important to consider the nature of this phenomenon, of the rise of RRI, because it appears to mobilise various actors and will already for that reason have effects (Fisher and Rip 2013). Our understanding will necessarily be partial, because we are in the midst of the process. Still, one can make an attempt, even if it will necessarily be somewhat essayistic. There have been studies and reports on what might be the substance of RRI and its potential implementation; a good overview is given in Owen et al. (2013a). I want to raise more distantiated sociological questions: what are the roots of this phenomenon? What are the dynamics at present, and what do these imply for the future of RRI as a discourse, and as an emerging patchwork of practices? Such questions are now being raised, for example in the volume edited by Owen et al. (2013a) and in a conference in Oslo, December 2012^b^. Papers deriving from that conference have been published in earlier issues of this journal, and are brought together in a Thematic Series entitled ELSA and RRI; this paper is the last in the series^c^.

My approach is to consider RRI as a social innovation that is gradually being articulated. What is being innovated are the roles and responsibilities of actors and stakeholders in research and innovation. Following Shelley-Egan (2011) and Rip and Shelley-Egan (2010), I will analyse this as a division of moral labour (an element in the overall cultural and institutional division of labour in societies), and position RRI in a historically evolving division of moral labour. This will then help me to trace the emerging path of RRI as a social innovation, and evaluate some of its features.

The historical-sociological approach is important to avoid limiting ourselves to a purely ethical perspective. I will introduce it briefly by comparing an earlier (16^th^ century) issue of responsibility of scientists with a recent case which shows similar features. Broader responsibilities of scientists have been on the agenda, definitely after the Second World War and the shock (in the sense of lost innocence of physicists) of the atom bomb and its being used^d^. Thus, there is a past to RRI, before there was the acronym that pulled some things together. I say “some things” because there is no clear boundary to issues of responsibility linked to science. As a sociologist, I think of it as an ongoing patchwork with some patterns but no overall structure, where a temporary coherence and thrust can be created, now with the label RRI, which may then diverge again because patchwork dynamics reassert themselves.

With the benefit of the extended analysis of divisions of moral labour, informed by the notion of a language of responsibility, I can address the emerging path of RRI, including the reductions that occur, inevitably. These reductions, and institutionalisation in general, are the reason to include some evaluation of future directions, and relate them to wider issues in the final comments.

### An Evolving Division of Moral Labour

Let me start with a historical case, and compare it with a recent one in which similar features are visible. The 16^th^ century Italian mathematician and engineer Tartaglia had to make a difficult decision, whether he would make his ballistic equation (to be applied to predict the trajectory of a cannon ball) public or not^e^.

In 1531 the Italian mathematician Nicola Tartaglia developed, inspired by discussions with a cannoneer from Verona whom he had befriended, a theory about the relation between the angle of the shot and where the cannon would come down. He thought of publishing the theory, but reconsidered: “The perfection of an art that hurts our brethren, and brings about the collapse of humanity, in particular Christians, in the wars they fight against each other, is not acceptable to God and to society.” So he burned his papers (he had told his assistant Cardano about his theory, and Cardano published it a few years later).

But he changed his position, as he described it in his 1538 book *Nova Scientia*. “The situation has changed, with the Turks threatening Vienna and also Northern Italy, and our princes and pastors joining in a common defence. I should not keep these insights hidden anymore, but communicate them to all Christians so that they can better defend themselves and attack the enemy.

Now move forward to a case from 2013. In the online version of the *Journal of Infectious Diseases*, October 7, Barash and Arnon published their finding of the sequence of a newly discovered protein, but without divulging the actual sequence. The news item about this in *The Scientist Magazine* of 18 October 2013 says:

[This] represents the first time that a DNA sequence has been omitted from such a paper. “Because no antitoxins as yet have been developed to counteract the novel *C. Botulinum* toxin,” wrote editors at *The Journal of Infectious Diseases*, “the authors had detailed consultations with representatives from numerous appropriate US government agencies.”

These agencies, which included the Centers for Disease Control and Prevention and the Department of Homeland Security, approved publication of the papers as long as the gene sequence that codes for the new protein was left out. According to New Scientist, the sequence will be published as soon as antibodies are identified that effectively combat the toxin, which appears to be part of a whole new branch on the protein’s family tree.

There are other cases where possible publication of sensitive details are prohibited, by the US National Science Advisory Board for Biosecurity, as in the case of the bird flu research by the Rotterdam team led by Fouchier (see also Evans and Valdivia, 2012).

My point here is about the similarities of the two cases, including the trope of powerful knowledge (at least, that is how the scientists and others see it), and how it can be used and misused. In the cases, the primary response to the possibility of misuse was to keep this knowledge hidden, but this will depend on the situation and the evolving balance of interests and visions. Whether to make such knowledge publicly available, and in fact, whether to invest in developing it at all, has to be evaluated again and again.

Thus, the structure of the considerations is the same, but the difference is that in the 21^st^ century, the decisions are not individual but part of formal and informal arrangements and authoritative decisions by advisory boards and government agencies. What is also interesting is that there is no reference to responsibility of the researcher/scientist. In the 16^th^ century this was because the word did not yet exist. In the 21^st^ century, it was because the focus is now on what is permissible and expected, rather than an own responsibility of the researchers. The division of moral labour has changed.

Before I continue to discuss present divisions of moral labour and how RRI can be positioned in that landscape, I need to briefly look at how the words ‘responsible’ and ‘responsibility’ have been used, and are still used, particularly to articulate roles and duties in an evolving social order, and then add how such roles can be part of long-term “settlements” of science in society (what is sometimes called a “social contract” between science and society, cf. Guston and Kenniston (1994)).

Elsewhere I have shown there is an evolving “language” of responsibility, in general and for scientists and scientific research (Rip 1981). The big dictionaries of modern languages (Oxford English Dictionary, Grande Larousse etc.) offer historical data on the use of words. The adjective (sometimes used as a noun, as in the French ‘responsable’) has been in use for a long time, in French since the 13^th^ century, in English since the 17^th^ century, but in a variety of meanings^f^. It is in the 18^th^ century that stabilisation occurs into the pattern of meanings that we see nowadays.

The noun “responsibility” is only used since the late 18^th^ century: since 1782 in French, since 1787 in English (those are the earliest quotes presented in the dictionaries). It is important to keep the relatively recent emergence of the term “responsibility” in mind because the term is often used to refer to thoughts and analyses in texts of pre-19^th^ century philosophers (e.g. Aristotle, Hume) who do not use the term. This suggests a continuity which is not there, and backgrounds societal developments through which the term “responsibility” emerged and obtained its meanings. But the sociogenesis of the concept of responsibility is not visible in handbooks and studies of morality in the past, because almost all authors tend to project present-day language usage onto the past^g^.

What happened at the turn of the 19^th^ century and stabilized in the course of that century is the emergence of bourgeois society and the idea of citizens (*citoyens*) with their rights and duties. To articulate those, an extension of language was necessary – the language of responsibility. Through that language, it became possible to discuss and fill in social order concretely. And some outcomes would find a place in the formal Constitution of the nation states as they organized themselves. This language of responsibility remains important to discuss evolving social orders, in the small and in the large. And it has become important for scientists (the term itself being an early 19^th^ century invention, see Ross 1962) and science^h^.

Before the notion of ‘responsibility’ had become important (and available at all) from the early 19^th^ century onward, relations between science and society could already be at issue, in particular as a relatively protected space in exchange for acquiescence to the existing order (Rip 2011). In retrospect, one can see that a long-term “settlement” between science and society started in the late 17^th^ century (in the 1660s in France and Britain, to be more precise), one indication being how the UK Royal Society was established with an implicit political charter: you can do science if you don’t interfere in society.

The Business and Design of the Royal Society is: To improve the knowledge of natural things, and all useful Arts, Manufactures, Mechanick practices, Engynes and Inventions by Experiments - (not meddling with Divinity, Metaphysics, Moralls, Politicks, Grammer, Rhetorick, or Logick)^i^.

It is clear that the Royal Society's founders avoided theology and politics; and in not meddling with "Grammar, Rhetorick, or Logick," the three basic disciplines of a university education, they also kept a distance between their "Business" and the universities.

This social contract between (emerging) science and society created a macro-protected space for science (Rip 2011), provided scientists showed prudential acquiescence to the powers that be^j^. Prudential acquiescence can actually be counteracted by a vision of progress through science which has served as a mandate for the autonomy of science, but could also lead scientists to become active in the wider world, as an embodied force of progress. This is quite visible in present newly emerging science and technology: scientists can speak for new and promising science (from astrophysics to cancer research) and for the importance of scientific approaches in improving the lot of mankind. Such messages can be taken up by others, and be further amplified (cf. below on narratives of praise and blame).

The overall settlement went through phases, with the ideal of an “ivory tower” coming into its own in the late 19^th^ century^k^, then broken open by claims of relevance (already in and after the first World War), and contestation about relevance from late 1960s onward. The emergence of RRI might indicate that a next phase of the settlement is emerging, or at least that there are openings for it to happen.

### Present issues (including RRI) in the division of moral labour

There are justifications involved in claims and routines about roles and responsibilities, explicitly but also when they are taken for granted. A common view was captured in an aphorism by Ravetz (1975): “Scientists take credit for penicillin, but Society takes the blame for the Bomb”. One can criticize such a view as gerrymandering, claiming the good and disavowing the bad (or at least, shifting the responsibility to others). But it is also a way of dividing labour (here, moral labour) to perhaps doing better overall. Think of the view that scientists have a moral obligation to work towards progress, and that is how they discharge their duty to society, while others (better qualified, or more responsible, or at risk) should look after social, ethical and political issues. What Ravetz’s aphorism brings out is that one might want to query such a division of moral labour. This is a second-order ethics question: why would this be a “good” division of labour? Second-order ethics discusses the ethical (and more broadly, normative, cf. Rip 2013) aspects that become visible when one inquires into the justification of present overall social and institutional arrangements, rather than taking them for granted.

Developments since the 1970s, and particularly in the 2000s, are undermining this common justification and that is the reason why notions like responsible development of new technologies and responsible research and innovation have emerged. RRI need not go for implementation and good practices only, but can include second-order ethics and be a focus point to discuss the pros and cons of this division of moral labour^l^.

What RRI does already is to reinforce trends towards stakeholder and citizen engagement with science, and to some extent, innovation. It is useful to consider division of moral labour again, now for other actors than scientists.

Let me start with the well-known industrialist’s argument about the need to go for profit to survive, while other actors should take care of second-order, possibly negative effects (most often, government actors are assumed to have this responsibility). While this argument continues to be heard, practices are different. The move towards corporate social responsibility is one example, and particularly important is the Responsible Care Program in the chemical sector (King and Lenox 2000). From a sociological perspective, one can see the importance of the notions of “good firms” and “cowboy firms” (or “rogue firms”, cf. notion of “rogue states” on the global scene). The “good firms” behave well, according to a division of moral labour, and are to be praised for their efforts even if outcomes are not always ideal. While “cowboy firms” transgress and must be condemned, particularly because they endanger the credibility of the “good firms” in the sector^m^.

Analysis in terms of division of moral labour can also be used to understand the actual and possible role of lay people, citizens, and consumers. Consumers, for example, are projected as having a duty to buy, and be informed, and calculate rationally – if only to ensure that neo-classical economics remains applicable. But they can also go for political action through consumption decisions, including boycotts (cf. Throne-Holst 2012). And there are evolving liability regimes which shift the responsibilities between producers and consumers (cf. Lee and Petts (2013), particularly p. 153).

The present interest in public engagement often remains within traditional divisions of moral labour by positioning members of the public as articulating preferences which may then be taken up in decision making as additional strategic intelligence. But one could have joint inquiry into the issues that are at stake (Krabbenborg 2013). In Codes of Conduct (as for nanotechnology) and broader accountability of scientists and industrialists generally, there is an assumption that there will be civil society actors willing and able to call them into account. That may not be the case: civil society actors may not be able, or not be willing, to spend the necessary time and effort. This is already visible in so-called “engagement fatigue”.

If one wants to overcome the traditional divisions of moral labour (for emancipatory reasons or because the present division of labour is not productive) other divisions of moral labour have to be envisaged and explored. One entrance point would be to consider evolving narratives of praise and blame (Swierstra and Rip 2007, Throne-Holst 2012) and turn them into blueprints of division of moral labour. This is a complex process, also because of the reference to possible future developments and the “shadow boxing” about the promises that ensues: Wonderful futures can be projected, waiting to be realised, which then justifies present efforts and allows criticism of those who don’t want to join in.

Compare this quote from Philip J. Bond, US Under-Secretary of Commerce, ‘Responsible nanotechnology development’, in SwissRe workshop, Dec 2004:

“Given nanotechnology’s extraordinary economic and societal potential, it would be unethical, in my view, to attempt to halt scientific and technological progress in nanotechnology. (…) Given this fantastic potential, how can our attempt to harness nanotechnology’s power at the earliest opportunity – to alleviate so many earthly ills – be anything other than ethical? Conversely, how can a choice to halt be anything other than unethical?”

What is not taken up in such sketches of a desirable world -- just around the corner, if only we would go forward without hesitation (in the quote, by pursuing nanotechnology) --, is the question of what makes these worlds desirable compared to other possibilities. It is a promise of progress, somehow, and when there is criticism, or just queries, rhetorics kick in. At the height of the recombinant DNA debate, second half of the 1970s, the medical possibilities were emphasized: “Each day we lose (because of a moratorium) means that thousands of people will die unnecessarily”. The justificatory argument about GMO, in the contestation about its use in agriculture, now refers to hunger in developing countries (which need biotechnical fixes, it appears). If the promise is contested, a subsidiary argument kicks in: people don’t understand the promise of the technology so we have to explain the wonders of the technology to them. (This is the equivalent of the well-known deficit model shaping exercises of public understanding of science.).

One sees here how narratives of praise and blame become short-circuited: only praise for the new technology is allowed. Some short-circuiting is inevitable, though. For example, hype about new technologies may be necessary to draw attention to them and mobilise resources for their further development – otherwise their promise would never materialise. Even while hype may lead to disappointment later on, as actors realise but apparently can’t do much about (Rip 2006, Rip 2013). At the same time, there might be concerns about the nature and impacts of the new developments, which again can be exaggerated in order to get a hearing. There is shadow boxing all around. Still, there are implications for a division of moral labour. For example, the importance of early warning is now widely recognized (cf. Harremoës (2001) on late lessons of early warning), but who can tell, at an early stage, whether a warning is significant? Mandates might become articulated for who may legitimately warn. Critical NGOs might be candidates for the task of voicing concerns and pushing them on the agenda; but this can also be viewed as unnecessarily harassing the promoters of technology. RRI will have to confront these issues in actual practice, and may try to articulate rules, procedures (beloved by bureaucracies), and new “good” practices (the quotes are used to indicate that one cannot stipulate beforehand what will be “good”).

Over time, there will be some solidification of divisions of moral labour, discursively, culturally, and institutionally. The emerging path of RRI as a social innovation is part of this broader institutionalization process. It will be shaped by it, but shape it as well, also by making it more reflexive.

### A path into the future

Arguably, the cradle of RRI as a social innovation were the concerns about nanotechnology in the early 2000s. Concerns in two senses: concerns from commentators and NGOS about possible negative impacts of the new and still uncertain nanotechnology, and concerns from promoters that nanotechnology would face lack of acceptance and active resistance as had happened with biotechnology – so this time, they should do it right from the very beginning^n^. Part of “doing it right” was to communicate better with various publics. Another part was to talk of responsible development of nanotechnology, and explore possibilities what this might imply.

In the Mid-Term Review of the US National Nanotechnology Initiative, there was an attempt to loosely define responsible development of nanotechnology:

Responsible development of nanotechnology can be characterized as the balancing of efforts to maximize the technology’s positive contributions and minimize its negative consequences. Thus, responsible development involves an examination both of applications and of potential implications. It implies a commitment to develop and use technology to help meet the most pressing human and societal needs, while making every reasonable effort to anticipate and mitigate adverse implications or unintended consequences. (National Research Council 2006:73).

This attempt at definition reflects a consequentialist approach (and ethics) as in traditional technology assessment. This strand continues in the articulation of RRI, but is not the only strand, particularly in Europe where the interest is also in inclusive governance of new science and technology^o^.

There are more such calls for responsible development, especially of nanotechnology. An interesting example are the meetings of the International Dialogue on Responsible Research and Development of Nanotechnology, positioned as opening up a space for broad and informal interactions (Tomellini and Giordani 2008, see also Fischer and Rip 2013), but hopefully, having consequences. In the first meeting in 2004, there was a proposal to develop a Code of Conduct, which was eventually taken up by the European Union (see European Commission 2008). Interestingly, the Code is much broader than the consequentialist ethics visible in the review of the US National Nanotechnology Initiative; see in particular the reference to a culture of responsibility (N&N stands for Nanoscience and Nanotechnologies):

Good governance of N&N research should take into account the need and desire of all stakeholders to be aware of the specific challenges and opportunities raised by N&N. A general culture of responsibility should be created in view of challenges and opportunities that may be raised in the future and that we cannot at present foresee (Section 4.1, first guideline).

Responsible development of nanotechnology, and the general idea of responsible innovation, have now become part of the policy discourse^p^. RRI is becoming an umbrella term, cf. the discussions leading to the European Commission’s Horizon 2020 Programme^q^, while scientists already start to strategically use RRI in funding proposals (and are being pushed to do so by EU policy officers), and ethicists see opportunities to expand their business (even if they may have moral qualms about its implications)^r^.

Branching out from responsible development of nanotechnology, and its precursor in the Human Genome Project’s ELSI component, and ELSA studies more widely, there is now also consideration of responsible synthetic biology and geo-engineering, with or without reference to RRI.

Clearly, RRI is an attempt at social innovation, ranging from discursive and cultural innovation to institutional and practices innovation^s^. As with technological innovation, a social innovation is new and uncertain, and distributed. Because of the many and varied inputs, the eventual shape of the innovation will be a *de facto* pattern, with dedicated inputs. To get taken up, institutional changes and sub-cultural changes (where different actors have to change their practices) are necessary. Such changes can be stimulated by soft command and control, as when in the EU (and Member states) codes of conduct for RRI would be stipulated. But it is also a business proposition: to extend the ‘social licence to operate’ because of credibility pressures in/of society. And now also a link with working on so-called Grand Challenges (e.g. Owen et al. 2013b).

Responsible research and innovation implies changing roles for the various actors involved in science and technology development and their embedding in society. This is an important aspect of the social innovation of RRI, and reinforces its embedding in an evolving division of institutional and moral labour in handling new technology in society^t^. An example is how technology enactors cannot just delegate care about impacts to government agencies and societal actors anymore, while it is not clear yet what a new and productive division of labour and its specific arrangements might be^u^. Thus, RRI opens up existing divisions of moral labour, concretely as well as reflexively (the latter in the sense that it positions them as not necessarily given a priori, but constructed and constructable).

One can inquire into the kind of arrangements that should be created (*ab novo* and/or by modulating existing and evolving arrangements). There is a patchwork of considerations and experiments already. Strong trends are to organize new types of stakeholder interactions^v^, and to have public engagement exercises as an input in development trajectories (Krabbenborg 2013)^w^. Background considerations include the struggle between consequentialist ethics and virtue and good-life ethics. Dominant is the utilitarian ethics perspective: maximize technology’s positive contributions and minimize negative consequences. And a neo-liberal version of it: it is enough if actors avoid causing harm. There is also the narrative of containment: keep hazards at bay, then there is no problem with a new technology, and enactors (developers and promoters who “enact” new technology, see Rip (2006) for the background to this terminology) can do what they want. The rise of ELSA studies and their equivalents functions as partial compensation for just pushing new (“promising”) technology, and thus legitimizes continued development.

These considerations and ongoing attempts will continue, and some will stabilize, contributing to an emerging path in the development of the social innovation of RRI. This has to do with entanglements, how actors refer to RRI and take it up in their repertoires, filling it in, and in particular ways. Inevitably, there will also be reductions, some productive, some less so, or perhaps just premature^x^.

In ongoing practices, whether these refer explicitly to RRI or not, we see reductions to create some tractability: a focus on upstream (to assure acceptance!?) – while the real challenges might be downstream. And a focus on risk issues – which appear to be more tractable than societal and ethical issues. These reductions can close down broader reflexivity, and definitely shape development, e.g. through evolving narratives of praise and blame. One example would the acceptance of versions of due process argument: “Was there upstream interaction with society? OK, enactor, then you cannot be blamed for what happens afterwards.”

Evolving paths become settled in institutional arrangements, and the new arrangements are taken as given, somehow. While this is understandable, given the need to act and make a difference, and thus reduce complexities to concrete activities (“Let’s get on, we’re doing responsible development, we’re OK”), it can be problematical, already because of the lock-ins (i.e. path dependencies) while society (and technology) change. One should ask “is this still a good arrangement?” This is not an argument for remaining open-ended in general, for its own sake, but to advocate monitoring the reductions that occur, and evaluating the directions that appear to be taken. There are no pre-given evaluation criteria, but the attempt, and the process, of such evaluations is important^y^.

### Final comments

The advent and further development of RRI (as a social innovation) is part of larger processes, is being shaped by them, and in a small way, contributes to them. It is creating openings in existing divisions of moral labour, not just of scientists and technologists, but also industrialists, government actors and society actors. That is to be be valued, and while some closing down is necessary, that should occur reflexively.

How is the RRI path shaping up, and how will it settle? The role of the European Commission, as a catalyst in multi-level dynamics (Fisher and Rip 2013), will be important^z^. Scientists will continue to be prudentially acquiescent, but under the RRI regime they will now more often be held to account^aa^. A consequence is that impact, or better, embedding in society, will be seen as part of professional responsibility of scientists, even if most often they cannot do much about it.

Industrialists are facing customers all the time. For newly emerging science and technology, these customers are often other businesses, while end users enter the picture only at one or two removes. With RRI, the responsibilities of industrialists are extended. One effect might be more interaction across the product-value chain.

NGOs and Civil Society Organisations come in as ‘third parties’, and are invited by the European Union and some government agencies to participate in responsible development of new technologies, even when they are not equipped (or willing) to do so (Krabbenborg 2013).

When these continue and stabilize, they add up to a master narrative for the further development of the social innovation of RRI. While there are explicit policy attempts (at least in the European Union) to create such an RRI path, what actually happens is an effect of ongoing struggles among many actors, and the possibility and desirability of such a path is one item in the struggles. Actually, one possible development is that RRI as a label for a policy fashion loses its force. But even then, some good practices will have evolved and remain. An example would be the emerging interest in extended impact statements when submitting proposals to funders, and some competence how to do them, and how to assess them.

Clearly, the “settlement” of science in society is changing, with the new discourse of RRI and the related practices being one element, reflecting these changes as well as pushing them. Does this amount to a shift in how our societies order themselves, at least with respect to newly emerging science and technology, which is similar to the emergence of responsibility language and practices in the early 19^th^ century? Not by itself, but it is part of a broader movement towards increasing social accountability of professionals and porousness of institutions which authors like Ulrich Beck have tried to capture with the notion of reflexive modernisation. While we need not follow Beck in his ideas about the necessary triumph of “second modernity” (Beck et al. 2003), the future of RRI is bound up with such larger changes, depending on them but also contributing to them.

### Endnotes

^a^See Barben et al. (2007) and Rip (2010).

^b^This was a dialogue conference, 4–5 December 2012, organized by the Oslo Research Group on Responsible Innovation, HiOA, located at the Oslo and Akershus University College for Applied Sciences, in cooperation with the Research Council of Norway’s ELSA programme. For more details, see Forsberg (2014).

^c^Earlier papers in LSSP, part of the Series on ELSA and RRI, are Myskja et al. (2014), Zwart et al. (2014), Oftedal (2014) and Forsberg (2014).

^d^As one response, the Bulletin of the Atomic Scientists was established in 1945. Kevles (1978): 335 quotes a leading physicist (James Franck) at the time as saying that scientists could “no longer disclaim direct responsibility for the use to which mankind … put their disinterested discoveries.” The development and use of the atom bomb was considered a watershed for mankind, particularly by German philosophers like Karl Jaspers and Günther Anders (see Van Dijk 1992).

^e^I base myself here on a Dutch text, Bos (1975), who refers to Charbonnier (1928) for the story. Since I follow his text quite closely, I have used indents, even if it is not a quote in the strict sense.

^f^The quotes in the Oxford English Dictionary suggest the meaning of ‘responsible’ was not stabilized, different authors could use it in their own way. “The Mouth large but not responsible (= correspondent) to so large a Body” (1698); “This is a difficult Question, and yet by Astrologie responsible (= capable of being answered)”. In the 17^th^ century, the German language uses ‘verantwortlich’ in the sense of ‘verantwort’ (Grimm 1956), similarly Dutch ‘verantwoordelijk’ (Woordenboek der Nederlandse Taal 131), In German, this use continues, in Dutch it disappeared from regular use in the course of the 19^th^ century (except for the use of ‘onverantwoordelijk’ in the sense of ‘onverantwoord’).

^g^This tendency is frustrating in handbooks like the Dictionary of the History of Ideas (Wiener 1973) in which one would expect some sensitivity for historical developments. For example, in the Lemma on “free will and determinism” (vol. II, pp. 239–240) a brief sketch is offered of Hume’s ideas, based on his Inquiry Concerning Human Understanding, Section VIII, using the terms “responsible” and “responsibility” all the time, while Hume himself speaks of “blameable” and “answerable” (and once of “accountable”). (Hume 1955, pp. 107–109). Somewhat of an exception is Adkins (1975) who limits the anachronism to his title, and emphasizes (in his introduction, p. 4) that moral responsibility is not an important concept for the Greeks (and does not occur as a term), because of their view of the world and society. It is only because of the Kantian turn, he claims, that a view of the world and society emerges in which “For any man brought up in a western democratic society the related concepts of duty and responsibility are the central concepts of ethics.” (p. 2).

^h^To avoid misunderstanding: I am not saying that this is the only meaning of responsibility. There is retrospective responsibility, visible in blaming and liability, and prospective responsibility, important because we are creating futures all the time (Rip 1981, Grinbaum & Grove 2013).

^i^Robert Hooke’s draft statutes (1663) of the Royal Society, quoted after Van den Daele (1978): 25. Van den Daele’s overall analysis has informed (and inspired) my argument here.

^j^The concept of ‘prudential acquiescence’ was introduced by Haberer (1969), p. 323, as a general feature of science. Rettig’s (1971) point that there are exceptions is correct; however these are indeed exceptions. In other words, the macro-protected space not only protects, but also confines.

^k^It could actually be applauded, as when a leading Dutch newspaper, *Het Nieuws van de Dag* (2 April 1908), referred to the world famous Dutch theoretical physicist J.D. van der Waals, and asked rhetorically whether anyone would get a slice of bread more because of the Van der Waals equations. No, but that is exactly why we appreciate the cultivation of science (Rip and Boeker 1975: 458).

^l^This need not be a one-sided critique of closed science. One consideration is that it is important to have the scientific endeavour be protected from undue interference. This is quite clear for the micro-protected spaces of laboratories and other sites of scientific work, and the meso-level protected spaces of scientific communities and peer review, although there is also opening-up, ranging from citizen science to criticism of scientific practices and the knowledge that is being produced (Rip 2011). Seen from the side of society, the scientific endeavour is legitimate as long as scientists deliver, both in terms of their producing what is promised (progress, even if this can interpreted in different ways) and their adhering to the normative structure of science (cf. the issues of integrity of science). This is a mandate which justifies the relative autonomy of science – a sort of macro-protected space.

^m^Interestingly, discussions about integrity of science and the occurrence of fraud have the same structure. Fraud is positioned as deviation from a general good practice, and done by “rogue scientists”.

^n^For the general observation, see Rip (2006). For the evocative phrase about doing it right from the very beginning, this summarizes the wording in Roco and Bainbridge (2001), p. 2, and was picked up on later, e.g. when presenting a risk framework for nanotechnology, developed in collaboration between the chemical firm Dupont and the USA NGO Environmental Defense Fund (Krupp and Holliday 2005).

^o^‘Inclusive governance’ was an important goal for the European Commission since at least the early 2000s (European Commission 2003). It is not limited to new science and technology.

^p^Stevienna de Saille (University of Sheffield), in her study of all documents pertaining to RRI (from the European Commission and others), concluded (personal communication) that the first occurrence of the term was in December 2007, to characterize the topic of a workshop with nanotechnologists and stakeholders, organized by Robinson and Rip 2007 (Robinson and Rip 2007). Robinson and I were picking up something that was in the air (while only half a year before, in an earlier attempt to organize such a workshop, we could not raise much interest among the members of the EU Network of Excellence Frontiers, our primary audience (Robinson 2010, p. 387–388)). We had not seen this term RRI used before, but thought of it to avoid a too narrow focus on risk issues in the workshop discussions. The later use of the phrase had other sources within the European Commission. I mention our invention of the phrase mainly to pinpoint when it had become “in the air”.

^q^As EU Commissioner for Research, Innovation, and Science Máire Geoghegan-Quinn phrased it in her opening speech for the EU Presidency Conference on Science in Dialogue, towards a European model for responsible research and innovation, Odense, 23 April 2012: ”*Horizon 2020 will support the six keys to responsible research and innovation…and will highlight responsible research and societal engagement throughout the programme*” (quoted from the official text handed out at the conference). Geoghegan-Quinn M. http://ec.europa.eu/commission_2010-2014/geoghegan-quinn/headlines/speeches/2012/documents/20120423-dialogue-conference-speech_en.pdf

^r^The European Commission included, at the end of its 7^th^ Framework Programme, Calls for background studies on RRI, to which ethicists, legal and governance scholars, and innovation studies scholars responded.

^s^One innovative element is the shift in terminology, from responsibility (of individuals or organized actors) to responsible (of research, development and innovation). The terminology has implications: who (and where) lies the responsibility for RI being Responsible? This may lead to a shift from being responsible to “doing” responsible development.

^t^The earlier division of labour around technology is visible in how different government ministries and agencies are responsible for “promotion” and for “control” of technology in society (Rip et al. 1995). There is more bridging of the gap between “promotion” and “control”, and the interactions open up possibilities for changes in the division of labour.

^u^The reference to ‘productive’ is an open-ended normative point, a Kantian regulative idea as it were. It indicates that arrangements (up to the de facto constitution of our technology-imbued societies) may be inquired into as to their productivity, without necessarily specifying beforehand what constitutes ‘productivity’. That will be articulated during the inquiry.

^v^Cf. Constructive TA with its strategy-articulation workshops (Robinson 2010), where mutual accommodation of stakeholders (including civil society groups) about overall directions occurs – outside regular political decision-making.

^w^In both cases, traditional representative democracy is sidelined. This may lead to reflection on how our society should organize itself to handle newly emerging technologies, with more democracy as one possibility. There have been proposals to consider technical democracy (Callon et al. 2009) and the suggestion that public and stakeholder engagement, when becoming institutionalized, introduce elements of neo-corporatism (Fisher and Rip 2013: 179).

^x^In an earlier article in this series, Zwart et al. (2014) emphasize that in RRI, compared with ELSA, “economic valorisation is given more prominence”, and see this as a reduction, and a reduction they are concerned about. However, their strong interpretation (“RRI is supposed to help research to move from bench to market, in order to create jobs, wealth and well-being.”) appears to be based on their overall assessment of European Commission Programmes, rather than actual data about RRI. I would agree with Oftedal (2014), using the same references as he does, that the emphasis is on process approaches in which openness, transparency and dialogue are important.

^y^With RRI becoming pervasive in the EU’s Horizon 2020, and the attendant reductions of complexity, this is a concern, and something might be done about it in the sub-program SwafS (Science with and for Society). See http://ec.europa.eu/research/horizon2020/pdf/work-programmes/science_with_and_for_society_draft_work_programme.pdf

^z^The European Union’s activities are more than creating funding opportunities, there can be effects in the longer term. The Framework Programmes, for example, have created spaces for interactions across disciplines and countries, and particularly also between academic science, public laboratories and industrial research, which are now generally accepted and productive. The emergence of these spaces has been traced in some detail for the programmes BRITE and ESPRIT in the early 1980s, by Kohler-Koch and Edler (1998).

^aa^This does not just derive from the RRI regime. Generally, the protected spaces for science are opening up. With the call for relevance since the 1970s, spokespersons (including sponsors of science) for possible clients have become involved, for example ‘industry’, or ‘sustainability’. Another new development is that citizens are becoming active in knowledge production and even some quality control, with attendant deprofessionalization of scientific knowledge production.
